# How do South African policies address provision of contraception among adolescents?

**DOI:** 10.4102/phcfm.v16i1.3966

**Published:** 2024-07-08

**Authors:** Thabile J. Ketye, Gbotemi B. Babatunde, Olagoke Akintola

**Affiliations:** 1 School of Public Health, Faculty of Community and Health Sciences, University of the Western Cape, Cape Town, South Africa

**Keywords:** adolescents, contraception, health policy, sexual and reproductive health, South Africa

## Abstract

**Background:**

The South African government has pioneered several policy documents that emphasise the importance of sexual and reproductive health (SRH).

**Aim:**

We examined how national policies address access and provision of contraception to adolescents in South Africa.

**Setting:**

South African national policies.

**Methods:**

We systematically searched various academic databases such as EbscoHost, Science Direct, Google Scholar, PubMed and Scopus, and other relevant sources to obtain 854 policy documents. Using a set of explicit inclusion criteria, we screened and selected 11 South African policies for analysis. Next, we analysed three international policies and frameworks to extract the key elements from them. Thereafter, we used these key elements to develop an analytical framework for conducting the analysis of the South African national policies.

**Results:**

We found that South Africa’s SRH policies largely address the provision of contraception by following international guidelines. These policies recognise the value of providing contraception to adolescent girls. However, we also found gaps in some policies, which could impede how they are translated into practice. These include recognising that adolescent boys can play a role in contraception; adolescents have varying SRH needs and are a key stakeholder not only for policy development but also for monitoring and accountability.

**Conclusion:**

With a specific focus on South Africa’s contraception services in the public sector, these findings are relevant to policymakers, providers and users of contraceptives.

**Contribution:**

This review proposes recommendations that will assist with strengthening health policy development and thus improve primary health care services related to contraception for adolescents.

## Introduction

Unintended or unwanted pregnancy and sexually transmitted infections (STIs) are critical global public health issues.^1,2^ Previous studies^2,3^ show that more than 10 million adolescent girls have unintended births yearly in low- and middle-income countries (LMICs) in Africa, Latin America, the Caribbean, Asia and the Pacific regions. Globally, adolescents have varying sexual and reproductive health (SRH) experiences. However, adolescents in LMICs encounter more challenges such as pressure to get married or some form of forced sexual contact.^4,5^ Of an estimated 21 million girls aged 15–19 years who become pregnant, approximately 12 million of them give birth every year^3^ while the remaining proportion may be attributed to miscarriages and terminations.^2,3^ In sub-Saharan Africa, young women are among the key populations most vulnerable to SRH risks such as unplanned pregnancy and STIs, including the human immunodeficiency virus (HIV).^6,7^ Of the 13 million adolescent girls with an unmet need for contraception in LMICs, more than 30% live in sub-Saharan Africa.^4^

While adolescents are often considered healthy, research demonstrates that many die prematurely.^3^ Risky sexual behaviours predispose adolescents to adolescent-related SRH problems (such as HIV, STIs and unwanted pregnancies). Complications from pregnancy and childbirth are the leading causes of death among adolescent girls (ages 15–19 years).^8,9^ Adolescent mothers face higher risks of eclampsia, puerperal endometritis and systemic infections than women aged 20–24 years.^10^ Further, babies of adolescent mothers face higher risks of low birth weight, preterm delivery and severe neonatal conditions.^2,10^ Some adolescent girls opt for abortion or termination of pregnancy (ToP). However, of the 5.6 million abortions that occur globally each year in this age group, 3.9 million are unsafe and contribute to maternal mortality, morbidity and lasting health problems.^3^

Access to contraception and STI prevention interventions offers social, economic and health benefits.^11^ It is therefore imperative to have policies that promote non-discriminatory provision of contraception and STI prevention methods. Policies can influence the delivery of SRH services and enable access to contraception methods that can prevent unplanned pregnancy for adolescents.^12^ In an attempt to improve SRH among young people, the International Conference on Population and Development (ICPD) emphasised the need to offer SRH information and services to adolescents.^13^ The conference was a milestone that emphasised the importance of achieving universal access to SRH to advance national development goals. Universal access to SRH services, including access to contraception, is an important aspect of the United Nations (UN) Sustainable Development Goals (SDGs) 2030. Sustainable Development Goal 3 aims to ensure good health and well-being at all ages and the provision of SRH services. Twenty-five years after the first conference, advancing the goals of the ICPD and safeguarding rights and dignity for all remain at the core of SRH interventions.^13^

Recently, the South African government published a number of policy documents that emphasise the importance of SRH. These include the National Clinical Guideline for Implementation of the *Choice of Termination of Pregnancy Act* (CToP),^14^ the National Integrated Sexual and Reproductive Health Rights (SRHR) Policy^15^ and the National Adolescent and Youth Health Policy.^16^ South Africa’s SRH policies are enshrined in the country’s constitution,^17^ which recognises the human rights of citizens. Further, they are aligned with regional and international charters, including the 1994 ICPD Programme of Action,^18^ 1995 Beijing Fourth Conference on Women Declaration,^19^ the UN SDGs, and the 2006–2015 Southern African Development Community (SADC) SRH strategy^20^ and 2006 Maputo Plan of Action.^21^

We assessed how existing national policies address adolescent contraception in the South African public sector. In doing this, we followed the work of Hoopes et al.^22^ who utilised the World Health Organization (WHO) human rights framework to assess two South African contraception policy documents. The study^22^ found that the 2012 policy documents are comprehensive and forward-looking in providing normative guidance. However, there remain gaps in these policies that may create barriers for adolescents to access contraceptive services. Similarly, Cordova-Pozo et al.^23^ examined how Paraguayan laws and regulations addressed adolescents and their contraception education and service needs by using the WHO human rights analytic framework. With the SDGs in place, little is known about how current national policies address issues related to access and provision of SRH services to adolescents in South Africa. Thus, the aim of this article is to examine how South African national policies address issues relating to access and provision of contraception to adolescents.

## Research methods and design

We used document analysis to assess how existing national policies address adolescent contraception. Document analysis is a systematic procedure for reviewing or evaluating printed and electronic material.^24,25^ It is an essential component of health policy analysis that enhances procedural rigour and allows for a fuller understanding of policy processes and contents.^26,27^ In conducting the document analysis, we followed Matlapeng et al.^28^ (p. 167).

### Search strategy

We developed a search strategy to help us retrieve as many documents as possible that are relevant to the topic of our study. In developing the search strategy, we itemised and grouped various words to create broad search terms. For example, we used various key terms related to population group (i.e. adolescent OR teenager OR youth OR young people); pregnancy prevention (i.e. ‘family planning’ OR ‘contraception’ OR ‘contraceptive’ OR ‘birth control’ OR ‘population control’ OR ‘fertility control’), SRH service provision (i.e. ‘sexual health’ OR ‘reproductive health’) and policy documents (i.e. health policy OR ‘strategy’ OR ‘plan’ OR ‘framework’ OR ‘guideline’ OR ‘checklist’). Further, we used singular and plural terms to ensure exhaustive search results. This enabled us to retrieve all possible policy documents related to our topic.

**FIGURE 1:**
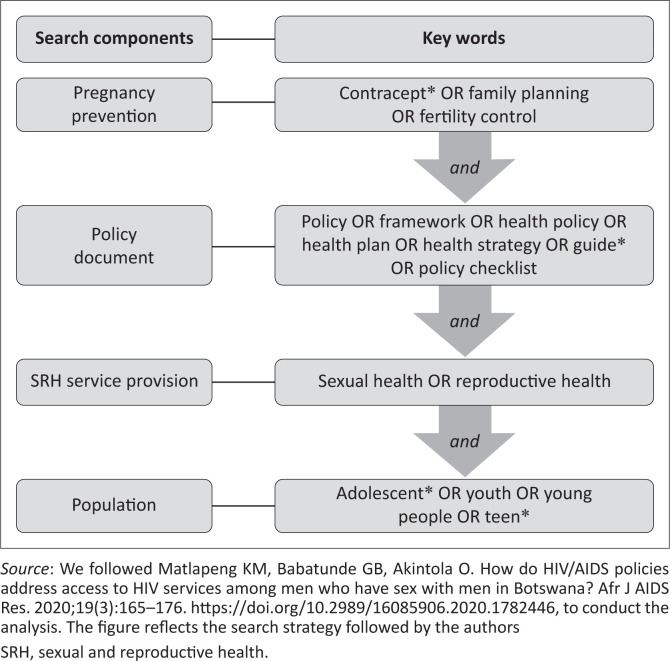
Search strategy.

Using key terms, we searched academic databases and other relevant websites to obtain relevant documents. The academic databases we searched include EbscoHost, Science Direct, Google Search and Google Scholar, PubMed and Scopus. Other sources were websites that host the following international and regional organisations or institutions: the African Union (AU), SADC, the United Nations Population Fund (UNFPA), the United States Agency for International Development (USAID) and WHO. In addition, we searched other web-based sources. We started by searching the Department of Health website (The Knowledge Hub) and then moved on to search other South African government departments’ (including the South African government) websites. The search of terms led to the identification of 4669 results or documents (see Figure 1 for the visual depiction of the search strategy).

**FIGURE 2:**
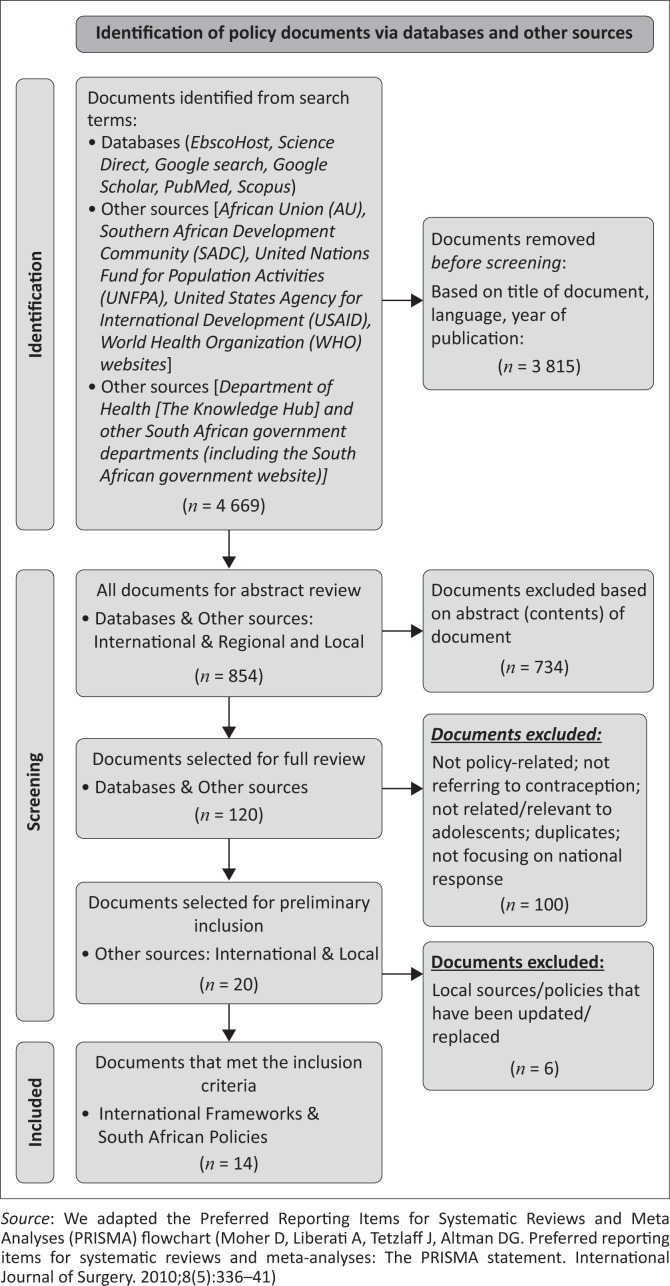
Flowchart showing the process of collecting and selecting policy documents for review.

Following this, we developed inclusion criteria that enabled us to retrieve relevant policy documents and thereafter applied the inclusion criteria systematically (see Figure 2). Specifically, the inclusion criteria were that the document had to be (1) international key policy documents that provide guidance to national policies on matters related to universal access to comprehensive SRH services for adolescents, (2) international key policy documents that serve as a guide for national policies on matters related to provision of contraception and/or STI prevention methods among adolescents, (3) national policy documents related to access or provision to contraception that have been adopted (for implementation) by the South African public sector and (4) national policy documents on SRH addressing contraception and/or STI prevention methods among adolescents in the South African public sector.

We excluded the following: (1) research articles and reports (documents that are not policies, frameworks, guidelines, or plans), (2) legislation, regulations, treaties and conventions, (3) policy documents with no reference to (both) adolescents and contraception (e.g. the Amended National Policy Framework on Child Justice^29^), (4) national policy documents adopted or implemented in other countries (outside of South Africa), (5) policy documents developed before South Africa became a democratic country in 1994 and (6) documents that are not written in English. This led to a total of 4669 results. At this point, we excluded 3815 documents based on the title, language (only documents written in English were included) and date of publication of each document.

### Screening and selection process

We conducted a three-phased screening approach.^30^ In Phase 1, we screened 854 documents by looking closely at the abstract of each article to ensure the selected document met the inclusion criteria. We then excluded documents based on the content of the abstracts. This left us with 120 documents. In Phase 2, we conducted a full review of each document to determine if the documents focused on contraception and/or SRH for adolescents or teenagers or young people. Thus, documents that are not policy-related, not referring to contraception, not related or relevant to adolescents and not focusing on a national response were excluded. In addition, we excluded duplicates. This led to Phase 3. In the third phase, we went through the 20 policy documents and excluded six policy documents that had been updated or replaced by more recent policy documents.

The following six policies (Box 1) were updated by the National Department of Health.

BOX 1 Policies were updated by the National Department of Health.

2015 policy documents:

- Department of Social Development & Department of Health. 2015. National Adolescent SRHR Framework Strategy, 2014–2019. This policy document was updated in 2019.
- Department of Health. 2015. National Consolidated Guidelines for the Management of HIV in Adults, Adolescents, Children and Infants and Prevention of Mother-to-Child Transmission. This policy document was updated in 2020.

2012 policy documents:

- Department of Health. 2012. National Contraception and Fertility Planning Policy & Service Delivery Guidelines (update of 2001 policy). This policy document was updated in 2019.
- Department of Health. 2012. National Contraception Clinical Guidelines. This policy document was updated in 2019.

2001 policy documents:

- Department of Health. 2001. National Contraception Policy Guidelines – within a Reproductive Health Framework. 2001. This policy document was updated in 2019.
- Department of Health. 2001. Policy Guidelines for Youth and Adolescent Health. The revised version was first published as a draft in 2012 and finalised in 2017.

A total of 14 (three international frameworks and 11 local) policy documents met the inclusion criteria and were therefore included in the subsequent analyses (Figure 2^28,48^ and Table 1).

**TABLE 1:** Policy documents for content analysis.

Contraception policy documents	
International frameworks	South African policies
WHO and UNFPA, 2015. Ensuring human rights within contraceptive service delivery: implementation guide. WHO, 2014. Ensuring human rights in the provision of contraceptive information and services: guidance and recommendations. United States Agency for International Development (USAID), 2014. Policy checklist: essential elements for successful family planning policies.	**2021** 1. Department of Health, 2021. South African National Integrated Men’s Health Strategy 2020–2025. 2. Department of Health, 2021. National Clinical Guideline for Implementation of the Choice of Termination of Pregnancy Act. 3. Department of Basic Education, 2021. Policy on the Prevention and Management of Learner Pregnancy in Schools (*after the 2018 draft*). 4. National Youth Development Agency, 2021. Integrated Youth Development Strategy (IYDS) Draft 2024/25. **2020** 5. Department of Health, 2020. National Consolidated Guidelines for the Management of HIV in Adults, Adolescents, Children and Infants and Prevention of Mother-to-Child Transmission (*updated version of 2015*). **2019** 6. Department of Health, 2019. National Integrated SRHR Policy Ed.1 (*update of 2011 policy and Department of Social Development and Department of Health 2015 Framework Strategy*). 7. Department of Health, 2019. National Contraception Clinical Guidelines (*update of 2012 and 2001 policies*). **2017** 8. South African National AIDS Council (SANAC), 2017. National Strategic Plan for HIV, TB and STIs 2017–2022. 9. Department of Health, 2017. National Adolescent and Youth Health Policy (*update of 2012 draft and 2001 policy*). 10. Department of Basic Education, 2017. National Policy on HIV, STIs and TB for Learners, Educators, School Support Staff and Officials in all Primary and Secondary Schools in Basic Education Sector. **2012** 11. Department of Health and Department of Basic Education, 2012. Integrated School Health Policy.

*Source:* We followed the approach used by Matlapeng KM, Babatunde GB, Akintola O. How do HIV/AIDS policies address access to HIV services among men who have sex with men in Botswana? Afr J AIDS Res. 2020;19(3):165–176. https://doi.org/10.2989/16085906.2020.1782446, to conduct the analysis

WHO, World Health Organization; UNFPA, United Nations Population Fund.

### Analysis of South African policy documents

Before looking at the 11 South African policy documents, we went through the three international frameworks. The three international frameworks (USAID,^31^ WHO,^32^ and WHO and UNFPA^33^) we chose met the inclusion criteria and provide guidelines that may be integrated in national policies. The USAID framework is a checklist of ‘essential elements for successful family planning policies’.^31^ The WHO framework has nine recommendations and 21 sub-recommendations grounded in human rights.^32^ The WHO and UNFPA (2015) guideline is an implementation guide of the WHO framework. The guideline translates the recommendations and sub-recommendations into nine concrete actions for midlevel policymakers and programme managers that are involved in providing family planning services.

We conducted thematic analysis to review the three international frameworks. The aim of the review was to identify the various themes that constitute key elements in the policy documents. The analysis followed a systematic process that began a review of relevant literature and policy documents. From this review, we identified five thematic areas which are listed as follows:

1. Accessibility to comprehensive contraception information and services. This includes eliminating (financial and eligibility) barriers to contraceptive provision and providing SRH services without mandatory parental or guardian authorisation and notification.
2. Meaningful participation by relevant stakeholders (and role-players in the policy development process). This involves active and informed engagement of individuals in the policy-making process.
3. Costed policy with clear monitoring and accountability plans. This entails reference to the policy costs or budget and monitoring and accountability plans.
4. Ensuring clinical guidelines and service standards for contraception services (reflecting WHO standards, current thinking and evidence). This relates to providing quality care, acceptability, respect of privacy and confidentiality, and the provision of scientifically accurate comprehensive sexual education programmes and comprehensive, evidence-based information (to ensure informed decision-making for users).
5. Non-discriminatory availability of all types of contraceptive methods. This relates to contraceptive security. Contraceptive security ensures that individuals can choose, obtain and use high-quality contraceptives whenever they need them.^32,33^ It is a key component of sustainable family planning or reproductive health services.

Next, we conducted a comprehensive analysis of the included South African policy documents by checking if they cover the key elements derived from our review of from the international frameworks. We developed a table that contains a list of all the documents. The titles of the South African policy documents were listed alongside the key elements of the international frameworks in a tabular format (Table 1). We reviewed each national policy document to determine if they included the identified key elements of the international frameworks. Thus, we collected and classified the data according to the five broad themes derived from the international frameworks.

Table 2 shows the South African policies that have been included for analysis and the thematic areas from the international policy documents.

**TABLE 2:** Titles and themes of selected policy documents.

South African policies		Themes or key elements								
		Accessibility to comprehensive contraception information and services	Meaningful participation by relevant stakeholders	Costed policy with clear monitoring and accountability plans		Ensuring clinical guidelines and service standards for contraception services				Non-discriminatory availability of all types of contraceptive methods
				Costing/budget	Monitoring and accountability plans	Quality of care	Acceptability	Informed decision-making	Respect of privacy and confidentiality	
1.	DoH, 2021. South African National Integrated Men’s Health Strategy 2020–2025†	✓	✓	reference unrelated to contraception	✓	✓	x	✓	✓	✓
2.	DBE, 2021. Policy on the Prevention and Management of Learner Pregnancy in Schools	✓	✓	✓	✓	✓	✓	✓	✓	✓
3.	NYDA, 2021. Integrated Youth Development Strategy (IYDS) Draft 2024/25	✓	✓	✓	✓	✓	✓	x	x	✓
4.	DoH, 2020. National Consolidated Guidelines for the Management of HIV in Adults, Adolescents, Children and Infants and PMTCT	X	In foreword only	x	✓	In relation to HIV	✓	✓	Confidential HIV counselling	✓
5.	DoH, 2019. National Integrated SRHR Policy Ed.1	✓	✓	✓	✓	✓	✓	✓	✓	✓
6.	DoH, 2019. National Contraception Clinical Guidelines‡	✓	✓	x	x	✓	✓	✓	✓	✓
7.	DoH, 2019. National Clinical Guideline for Implementation of the Choice of ToP Act Ed.1	✓	✓	x	✓	✓	✓	✓	✓	✓
8.	SANAC, 2017. National Strategic Plan for HIV, TB and STIs 2017–2022	✓	✓	in relation to TB and HIV	✓	x	✓	x	✓	✓
9.	DoH, 2017. National Adolescent and Youth Health Policy	✓	✓	✓	✓	x	✓	x	✓	✓
10.	DBE, 2017. National Policy on HIV, STIs and TB for Learners, Educators, School Support Staff and Officials in all Primary and Secondary Schools in Basic Education Sector	✓	✓	✓	✓	x	✓	✓	(broadly, not specific to contraception or adolescents)	✓
11.	DoH and DBE, 2012. Integrated School Health Policy	✓	✓	✓	✓	broadly covers quality of services	✓	refers to consent and assent for school health programmes	x	✓

*Source:* We adapted the approach used by Matlapeng KM, Babatunde GB, Akintola O. How do HIV/AIDS policies address access to HIV services among men who have sex with men in Botswana? Afr J AIDS Res. 2020;19(3):165–176. https://doi.org/10.2989/16085906.2020.1782446

† , No reference to contraception. Refers to adolescents and SRH.

‡ , The 122 page revised the National Contraception Clinical Guidelines (2019) and the associated South African Handbook for Contraceptive Method Provision (2019) build on two previous policy and guideline documents, the National Contraception and Fertility Planning Policy and Service Delivery Guidelines (2012) and the National Contraception Clinical Guidelines (2012).

**Key**:

✓, The themes or key elements are covered or included in the policy document.

X, The themes or key elements are missing or not included in the policy document.

**FIGURE 3:**
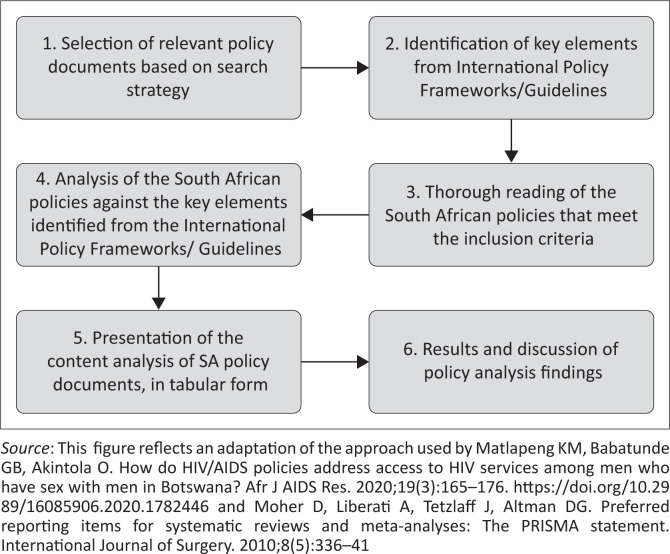
Policy analysis process.

We have summarised the overall findings of the policy analysis, based on the identified thematic areas, in the next section. See Figure 3^28,48^ for the policy analysis process.

### Ethical considerations

Ethical clearance to conduct this study was obtained from the University of the Western Cape Biomedical Research Ethics Committee (No. BM19/1/24).

## Results

We used five thematic areas that we obtained from the international frameworks to assess the current South African policies that are related to contraception and/or SRH prevention methods (as shown in Table 2). Overall, we found that each policy document focuses on its objectives and target group. As such, the policy documents complement each other and refer to other policy documents that may be of interest or that may be required to address a specific issue in detail.

### Accessibility to comprehensive contraception information and services

Except for the National Consolidated Guidelines for the Management of HIV in Adults, Adolescents, Children and Infants and Prevention of Mother-to-Child Transmission (PMTCT),^34^ almost all (10 out of 11) of the included policy documents broadly highlight the need to reduce any form of barrier to access to contraceptive information and services for adolescents and young people. The 10 policy documents outline barriers to accessing comprehensive contraception information and services. These include financial cost of contraception services, poor knowledge or lack of awareness, consent by a parent or guardian and distance to the service site.

On the other hand, the South African National Integrated Men’s Health Strategy 2020–2025^35^ aims to provide comprehensive and integrated care for men and boys but does not speak specifically to contraception, family planning or fertility control in relation to adolescents. It states barriers to accessing healthcare services (in relation to males), including the availability of options for males to discuss SRH issues with a male provider. The strategy states the following:

Several individual and health system factors prevent men from accessing health facilities. These include the availability of services, skill and capacity of health workers and the way services are offered. In addition, men’s personal factors, including stigma, poverty, feelings of compromised masculinity, confidentiality concerns, distance to the facility, inconvenient hours, and prevailing gender norms (perceptions that facilities provide women-centred services) also serve as barriers for men accessing services. (p. 8)

The strategy^35^ further states that:

The impact of unsafe sexual practices on the sexual health and wellbeing of adolescent males and their partners is an important consideration. Education about safer sexual practices is a critical intervention for the sexual health of adolescent boys, coupled with easier access to condoms for adolescents. (p. 11)

The National Integrated SRHR Policy,^15^ National Contraception Clinical Guidelines^36^ and National Clinical Guideline for Implementation of the *CToP Act*^14^ emphasise the need for access to family planning services. These policy documents note that adolescents are not a homogeneous group, and their SRH needs vary while considering factors that might serve as barriers to accessing the services.

In South Africa’s public health sector, contraceptive services are provided at no cost to users.^36,37^ Thus, the cost of contraception and STI prevention methods is not a deterrent of accessibility to contraceptives and condoms. In addition, the policy documents do not use words that are restrictive or have the potential of being coercive in practice. Thus, any adolescent can access contraceptives if they choose to, and they can do so without being forced. For example, the Integrated Youth Development Strategy (IYDS) Draft^38^ and National Adolescent and Youth Health Policy^16^ promote equal access to family planning and integrated SRH services among adolescents. However, the language that is used in practice might be different.

The Integrated School Health Policy^39^ discusses the importance of providing young people with preventive health information to improve their knowledge and level of awareness. In line with the *South African Children’s Act*,^40^ the National Policy on HIV, STIs and TB^41^ states that adolescents do not require parental approval to receive sexuality education.

### Non-discriminatory availability of all types of contraceptive methods

All the included policy documents mention contraceptive methods but to a varying degree. Some policy documents provide a list of the types of contraceptive methods that should be provided in public health facilities. Others merely mention the term ‘contraceptive’. In cases where detail is not provided, reference is made to the policies that provide the necessary details. The policy documents recommend the availability of various modern contraceptives, provider capacity and the prevention of commodity stockouts at public health facilities.

All the policies identify male condom distribution as one of the key indicators of access to contraception and STI prevention methods. However, policy documents such as the South African National Integrated Men’s Health Strategy^35^ do not distinguish between male and female condoms. Thus, it can be assumed that the reference is to either male or both types of condoms. Further, there is no mention of other contraceptive methods and how these may be made available. On the other hand, the policies on the Prevention and Management of Learner Pregnancy in Schools^42^ and the National Policy on HIV, STIs and TB^41^ recognise the need to provide male and female condoms and refer learners to the Department of Health (DoH) for other contraceptive methods.

The IYDS Draft,^38^ which is written by the National Youth Development Agency (NYDA), states the importance of strengthening family planning. However, it does not elaborate on issues related to SRH. Yet the NYDA is mandated to mainstream youth issues into society and champion youth development with all sectors of society. Unlike other policy documents, the South African National Integrated Men’s Health Strategy only mentions the male condom – it does not mention other contraceptive methods and how those may be made available. This may give the impression that preventing unwanted pregnancies is the responsibility of female adolescents. Interestingly, the strategy notes that for males aged 20–34 years, it is important to ‘leverage their societal roles to help improve women’s health (SRH, including family planning) … and reduce STIs’ (p. 12). However, it does not outline how this can be done at that age or among adolescents.

The Integrated School Health Policy^39^ advocates for the provision of dual protection (that is, a barrier contraceptive like an oral pill or injectable used alongside another method such as a condom to reduce STI transmissions and prevent pregnancy). The National Strategic Plan (NSP) for HIV, TB and STIs^43^ refers to the provision of non-discriminatory access to a:

[*P*]ackage of SRH services (that) will include counselling on contraception and voluntary medical male circumcision (VMMC), provision of contraception and condoms, pregnancy testing, HIV testing services (HTS) and pre-exposure prophylaxis PrEP. (p. 16)

On the other hand, the National Clinical Guideline for Implementation of the *CToP Act*^14^ states that every individual who seeks ToP can access the service without undue delay and within 7 days from the first request to access those services. In addition, there is no age of consent or a minimum age to access ToP services. This service is provided within the first 12 weeks of pregnancy without giving reasons.

The National Consolidated Guidelines for the Management of HIV in Adults, Adolescents, Children, and Infants and PMTCT^34^ stipulate that health facilities are responsible for ensuring that there is a constant supply of essential medicines and commodities, which include contraceptive methods in line with the National Contraception Clinical Guidelines.^36^ The National Contraception Clinical Guidelines^36^ indicate specific modern methods for various age categories, including adolescents. In addition, the Guidelines provide a list of available contraceptives and STI prevention methods like the National Integrated SRHR Policy.^15^ These are the intrauterine contraception, for example, copper intrauterine device (IUD), hormonal intrauterine system (IUS), subdermal contraceptive implants, condoms (male and female), progestogen-only injectables, oral contraceptive pills, emergency contraception (EC) and voluntary sterilisation. This is noted as being seldom an appropriate method for adolescents or young adults because it is permanent and irreversible (p. 45).

### Meaningful participation by relevant stakeholders (and role-players)

Most (10 out of 11) of the policy documents acknowledge the role and value of relevant multisectoral stakeholders (including key stakeholders at national and provincial departments, frontline healthcare workers, technical partners, academics partners, non-governmental organisations [NGOs], civil society and private sector institutions) in policy development and implementation. In addition, they advocate for adolescents or learners. These documents include the South African National Integrated Men’s Health Strategy,^35^ the Policy on the Prevention and Management of Learner Pregnancy in Schools,^42^ the National Integrated SRHR Policy,^15^ the National Contraception Clinical Guidelines,^36^ the National Policy on HIV, STIs and TB^41^ and the Integrated School Health Policy.^39^ The National Strategic Plan for HIV, TB and STIs^43^ lists accountability partners to realise SRH objectives. However, their roles in relation to the provision of adolescent health or SRH services are not specified. The National Consolidated Guidelines for the Management of HIV in Adults, Adolescents, Children, and Infants and PMTCT^34^ only refer to stakeholders in the document’s foreword (to acknowledge those who participated in the process).

Some policies specify the importance of including young people in the policy development and implementation processes. The IYDS Draft^38^ states that ‘the NYDA must work with national DoH to develop the comprehensive policy and legislative framework; by making sure that young people are part of the consultative process towards its development’ (p. 86). Further, the IYDS Draft^38^ plans to implement a programme on adolescent SRHR, which addresses teenage pregnancies and risky behaviour in collaboration with the national DoH and other government departments (such as the Department of Basic Education (DBE) and Department of Social Development (DSD) (p. 90). The National Adolescent and Youth Health Policy^16^ was developed through youth engagement and expert stakeholders from government, civil society and other organisations. Thus, it can be regarded as the policy document that was developed by the youth for the youth.

### Costed policy with clear monitoring and accountability plans

The budget or implementation costs were not stipulated in any of the policy documents. However, this does not mean that South African policies do not have cost, monitoring and accountability plans. It could mean that policy budgets are located in business plans or other related documents.

Six of the 11 policies refer to the need for policy funding and clear monitoring and accountability plans for their realisation. These are the Policy on the Prevention and Management of Learner Pregnancy in Schools,^42^ the IYDS Draft,^38^ the National Integrated SRHR Policy,^15^ the National Adolescent and Youth Health Policy^16^ and the Integrated School Health Policy.^39^

The Policy on the Prevention and Management of Learner Pregnancy in Schools^42^ refers to monitoring through multisectoral collaboration. This includes a coordination role by a DBE Sub-Committee, reviewing policy implementation across the different spheres of the department and regular reporting thereof. The Integrated School Health Policy^39^ specifies that national and provincial budgets should be utilised for attaining additional staff/human resources. A few references are related to the cost implications for users of contraception methods but not for the implementers. The IYDS Draft^38^ recommends, ‘The NDoH should give NYDA a certain budget allocation … through the National Treasury’ (p. 111) for SRH-related programmes. The National Integrated SRHR Policy^15^ recognises the need for a comprehensive implementation plan, which includes effective systems for managing the flow of clients through a facility despite fluctuations in caseload to ensure information flows to the district levels for budgeting purposes. The National Adolescent and Youth Health Policy^16^ refers to costing models and financing for the policy and the budget allocation for the implementation of adolescent health programmes. The policy states that there will be continuous monitoring and evaluation (M&E) by stakeholders through M&E guidelines.

The South African National Integrated Men’s Health Strategy^35^ states that it is the responsibility of the DoH to ensure the availability of supplies, commodities and infrastructure needs (p. 35), even though this is stated broadly and not necessarily in relation to SRH or contraception. The Department of Health notes in the Strategy^35^ that it will only succeed when there is a clear commitment to implementation planning and taking an active approach to evaluating, monitoring and reporting. The National Policy on HIV, STIs and TB^41^ refers to the alignment and coordination of budgetary priorities with policy and operational activities and monitoring of policy implementation. The National Strategic Plan for HIV, TB and STIs^43^ entails the budget estimates for the implementation of the NSP over 3 years. There is a reference to targets, budget and accountability plans. However, the NSP focuses on HIV and TB-related programmes and interventions, not contraception.

The National Contraception Clinical Guidelines^36^ and the National Clinical Guideline for Implementation of the *CToP Act*^44^ are silent on the financial modalities for implementing and monitoring the guidelines. This could be attributed to the fact that this policy document is specifically targeted at providers and should be used in conjunction with other national policies.

### Ensuring clinical guidelines and service standards for contraception services

Four policy documents (Policy on the Prevention and Management of Learner Pregnancy in Schools,^42^ the National Integrated SRHR Policy,^15^ the National Contraception Clinical Guidelines^36^ and the National Clinical Guideline for Implementation of the CToP^14^) meet the clinical guidelines and service standards for contraception services. The clinical guidelines and service standards relate to the provision of quality of care, acceptability, respect of privacy and confidentiality, scientifically accurate comprehensive sexual education programmes and comprehensive evidence-based information (to ensure informed decision-making for users).

These policies are aligned to various international, regional and national policy documents and clinical guidelines. The National Contraception Clinical Guidelines^36^ provides a detailed account of how providers should ensure that adolescents are receiving quality and appropriate youth-friendly health services that promote informed decision-making – in line with international clinical guidelines. Key counselling points and issues to take into account when providing SRH services to adolescents and young women are detailed in the National Contraception Clinical Guidelines^35^ for ease of reference for providers. The National Clinical Guideline for Implementation of CToP^14^ aims to enable all ToP-seeking individuals to make informed decisions and ensure their human rights are respected, protected and fulfilled (p. 2). However, it might be useful to note that the terms confidentiality and privacy appear only once in the National Integrated SRHR Policy.^15^

Seven policy documents partially ensure clinical guidelines and service standards for contraception services. The South African National Integrated Men’s Health Strategy^35^ recognises global and local international guidelines. However, there is no linkage between this Strategy and policy documents on contraception (as it focuses on key health issues for men and boys). In addition, there is a broad reference to strengthening the health system’s capacity to provide quality appropriate preventive care for men and boys, utilisation of data for decision-making and ensuring there is privacy for males during consultations with a General Practitioner or Medical Officer. The National Strategic Plan for HIV, TB and STIs,^43^ the IYDS Draft^38^ and the Integrated School Health Policy^39^ in relation to the quality of care and acceptability support this principle. The National Adolescent and Youth Health Policy^16^ broadly mentions the principles of acceptability and upholding privacy (p. 7). At the core, it states that comprehensive SRH services must be tailored to the needs of adolescents and in line with the Integrated Policy on SRHR.^15^

The National Policy on HIV, STIs and TB^41^ broadly covers SRH but is not specific to contraception or adolescents, and the National Consolidated Guidelines for the Management of HIV in Adults, Adolescents, Children and Infants and PMTCT^34^ covers the quality of care in relation to HIV.

## Discussion

South Africa has made positive strides by developing policies that prioritise contraception and STI prevention and consider the needs of girls and women. We found that generally South Africa’s SRH policies address provision of contraception and STI prevention methods by following international guidelines and standards. These policies recognise the value of providing contraception and STI prevention methods to adolescent girls. However, we also found gaps in some policies, which could impede how they are translated into practice.

Shortage of resources such as a lack of essential medicine and treatment can discourage people from seeking SRH services in the public sector.^45^ The primary health care (PHC) Standard Treatment Guidelines and Essential Medicines List specifies which contraceptive methods are available in South African public health facilities.^46^ However, it is unclear in the policy whether this means that contraception methods are available to adolescents and are provided to them without any prejudice or judgement. What is clear is that there are various ways to ensure non-discriminatory access and accessibility to contraception services. This includes making various contraception methods available to adolescents without the consent of a guardian or parent, as found in previous research.^44^

Previous research identified common barriers to SRH services among adolescents.^47,48^ It is beneficial for adolescents to be provided with SRH information and services so that they are able to make informed decisions.^44^ In addition, attaining contraception and STI prevention methods and SRH services in a language they can understand and from a nearest PHC facility makes it convenient for them. We found that these factors are included in the South African policies. This finding is consistent with that of other studies.^5,10^

On the other hand, there are policies that have provisions to prevent providers from restricting contraceptive services based solely on age and coercing young users. However, we found that there is no distinction made between the different age groups – some policy documents do not specify age categories. Thus, the application of those policies is for anyone who is sexually active. Yet, adolescents may have unique biopsychosocial needs.^49^ Addressing the needs of adolescents would help ensure that adolescents have access to quality and non-judgemental SRH services, and that they are provided with contraception and STI prevention methods in an adolescent-friendly setting.

We also found that the focus tends to be on women, thus minimising men’s role in preventing unintended pregnancies, as found in other research.^50,51^ Male condom distribution as a contraception and STI prevention method is one of the key interventions for preventing unintended pregnancy and STIs. The introduction of the South African National Integrated Men’s Health Strategy 2020–2025 is a step in the right direction. The Strategy states that male partner involvement is critical in SRH and therefore partner access to SRH services should be increased through knowledge and education (p. 32). However, it does not discuss male involvement in preventing unintended pregnancies. This is clearly a missed opportunity. Preventing unintended pregnancies should not only be the responsibility of girls and women.^52^ In future, it may be useful for South African policies to articulate this clearly and include measures specific to male and female adolescents. Research in France and South Africa shows that engaging male partners in SRH services improves men’s knowledge and attitudes towards the use of contraception and STI prevention methods.^53,54^

Previous research that used the WHO framework to analyse South Africa’s 2012 policy and clinical guidelines from a human rights perspective found gaps in the participation of adolescents in programme development.^22^ This is similar to our findings. Our findings show that youth involvement (as service users) in policy development can be improved by actively engaging with young people. It is critical to involve adolescents as a key stakeholder in the process of developing policies for young people. Young people can also be involved in monitoring policy implementation and ensuring accountability. The NYDA, as an agency that champions youth development, can play an active role in this regard.

### Recommendations

Ensuring access and provision of SRH interventions such as contraceptives and STI prevention methods to adolescents requires a multisectoral approach. There is room for government departments, youth organisations and the private sector to work together to bring SRH interventions to young people in more innovative and effective ways. Young people need access to SRH interventions in ways that speak to them directly. Policy makers should therefore include all relevant stakeholders in the policy development process. Both adolescent girls and boys are critical role-players in the policy development space and in the prevention of unintended pregnancies. Policies should discuss issues related to the role of boys and men.

## Conclusion

This study assessed how the current South African national policy documents address SRH interventions for adolescents and/or young people. We explored how the policy documents address access to and provision of SRH interventions, particularly contraception and STI prevention methods to adolescents. Using five key elements that we extracted from the USAID, WHO and UNFPA policy frameworks, we demonstrated the strengths and gaps in South Africa’s SRH-related policies and the potential implications.

Our study contributes to existing literature by showing the value of national policies in enabling access and provision of contraception and STI prevention methods to adolescents. It also explores how South African national policies can be strengthened to facilitate implementation. However, further research is required to look at how these policies are translated into practice by providers.
